# Electronic, optical and sodium *K* edge XANES in disodium helide: a DFT study

**DOI:** 10.1038/s41598-023-44369-z

**Published:** 2023-10-09

**Authors:** R. D. Eithiraj

**Affiliations:** Division of Physics, School of Advanced Sciences, Vellore Institute of Technology, Chennai, TN 600 127 India

**Keywords:** Materials science, Physics

## Abstract

The ground-state properties of the disodium helide (Na_2_He) in the cubic structure was calculated using the WIEN2k package within GGA, LDA, and mBJ potentials. From our results, the GGA and LDA predict the material to be semiconductor, while mBJ predicts the material to be insulator. The calculated results from the electronic structure show that Na_2_He is a direct bandgap semiconductor. Excitonic properties were studied and the results provide Mott-wannier type excitonic behavior of the material. The optical properties for Na_2_He were studied and its application towards optoelectronic devices has been identified. Also, Na *K* edge x-ray absorption near edge structure (XANES) for Na_2_He were computed and discussed. To verify the possibility of formation 2D structure (monolayer) of this compound, phonon calculations were performed. The result indicates that the 2D phase for this compound is dynamically unstable.

## Introduction

Helium is the most common element in the universe after hydrogen and is found in regular stars and in gas plants in a sufficiently large form^1^. Helium has many desirable chemical properties, such as the strongest potential for ionization and zero electron affinity^2,3^ making this element chemically inert. Due to this nature, Helium does not bond to any other elements to form a stable compound. In recent times, several scientists have tried to search out a stable helium (He) compound. Van der Waals compounds such as He@H_2_O (H_2_O)_2_He^4^ and NeHe_2_^5^ are the only known stable helium compounds. For the first time, researchers foreseen a stable compound in which He bond to a metallic element (Na) at a pressure of 300 GPa forms Na_2_He compound in cubic phase^6^. Mechanical and Thermodynamic property for this compound were studied by Zahidur et al.^7^. Similarly, Phonon transport properties for the Na_2_He compound were studied by San-Dong GuO et al.^8^. While substantial progress has been made in technically explaining the physical properties of the compound Na_2_He, there is a scarcity of information regarding the electronic structure.

The interpretation of ionisation edges, especially the study of energy loss near edge structures (ELNES), and their relationship to physical properties of modern materials, is of increasing attention for electron microscopists working with a spectrometer or an imaging energy filter. Multiple scattering (MS) and band structure (BS) approaches are the two types of ab initio procedures utilised in electron energy loss spectrometry. MS operates in real time and is commonly used to compute X-ray absorption near edge structures (XANES) and ELNES. Durham and colleagues invented this approach on the single scattering method^9^. BS approaches are employed in reciprocal space and relies on density functional theory (DFT)^10,11^.

The density functional theory (DFT) is a well-known approach for calculating a material's electronic properties. The computation of ELNES may thus also be done using it. Despite being known that DFT is not meant for the computation of electronically excited states, it works well for calculating ELNES/XANES and low-loss spectra. DFT is a highly active area of new advancements, and as computer power increases, so does the level of complexity of the issues covered and the reliability of the computations^12^. Muller and colleagues used DFT for the first time in the late 1970s to calculate X-ray absorption spectra using a linearised augmented plane waves approach. Much effort has been done in the last two decades to compare observed spectra with calculations^13,14^.

Among the several programmes available for DFT computations, two are commercially accessible that allow for the calculation of ELNES (CASTEP and WIEN2k)^15–17^. The WIEN2k code has been used effectively for ELNES computations in a variety of situations. One intriguing practical use was its use for phase identification when using reference spectra was not possible due to phases being metastable which is done for Fe_3_ phases in a nanocrystalline magnetic materials^18^. The current study is to ensure the viability of the material towards application in optoelectronic devices. In this study, electronic calculations and optical properties using different exchange correlation functional were computed for the unit cell of Na_2_He. Also, XANES calculations were computed at ambient pressure.

## Computational details

**Figure 1:**
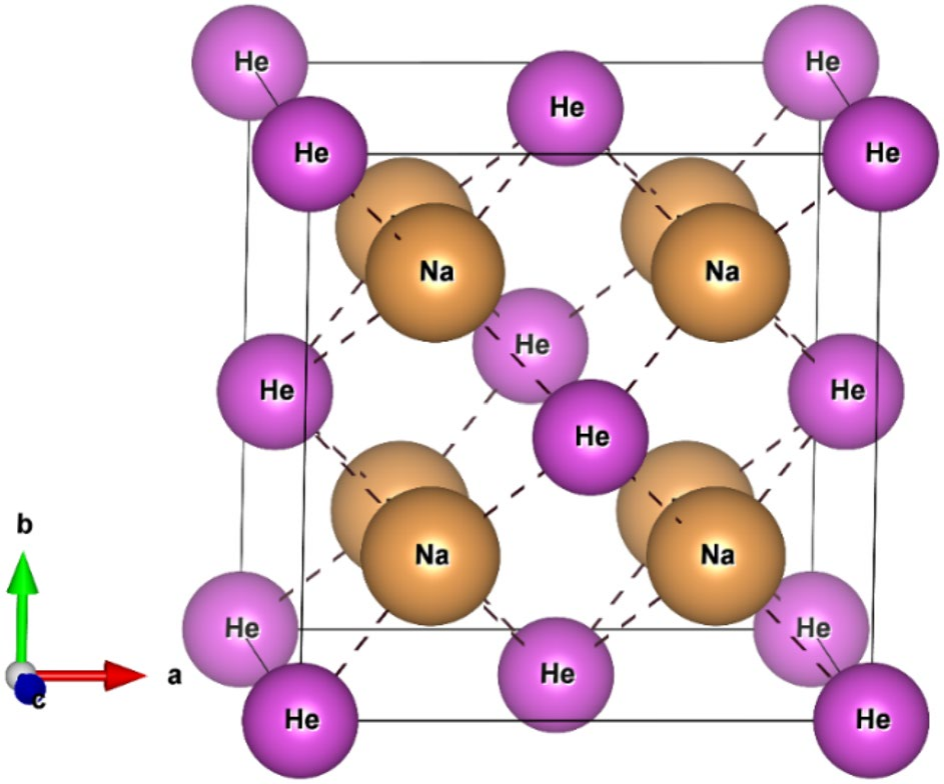
Crystal structure of Na_2_He compound.

In order to calculate the disodium helide electronic band structure and density of states (DOS) in the anit-CaF_2_-type structure, First-principles calculations were carried out using WIEN2k code^17^. In this methodology, electronic structure calculations were carried out within the GGA (PBE)^19^, LDA^20^ and mBJ potentials^21^ on the idea of the density functional theory (DFT)^10,11^. All the calculations were performed for the unit cell of Na_2_He. The RMT sphere radii for Na is 1.6 a.u. and He is 1.52 a.u. The basic structure of the antifluorite crystal (anti-CaF_2_) can be found in literature^22^. Because the structure of anti-fluorite CaF_2_ packing structure is low, an empty sphere is introduced in the position (0.5, 0.5, 0.5) position ± (0.25, 0.25, 0.25) and (0, 0, 0) without disrupting the crystal symmetry are occupied by the sodium and helium atoms. The RK_max_ value of 7 is given and the separation energy of − 6 Ry is given for the core-valence electron separation. For the calculation of all ground state properties a mesh of 413 k points was used. The Na_2_He structure is shown in Fig. 1. Additionally, x-ray absorption near edge structure (XANES) calculation were performed using 2 × 2 × 2 and 3 × 3 × 3 supercells along with 64 k points for Na* K* edge in Na_2_He. Moreover, for Na *K* edge the values of RK_max_ and L_max_ are fixed to be 8 and 10. The XANES calculations were done using GGA-PBE functional.

## Results and discussion

### Band structure and density of states (DOS)

**Figure 2:**
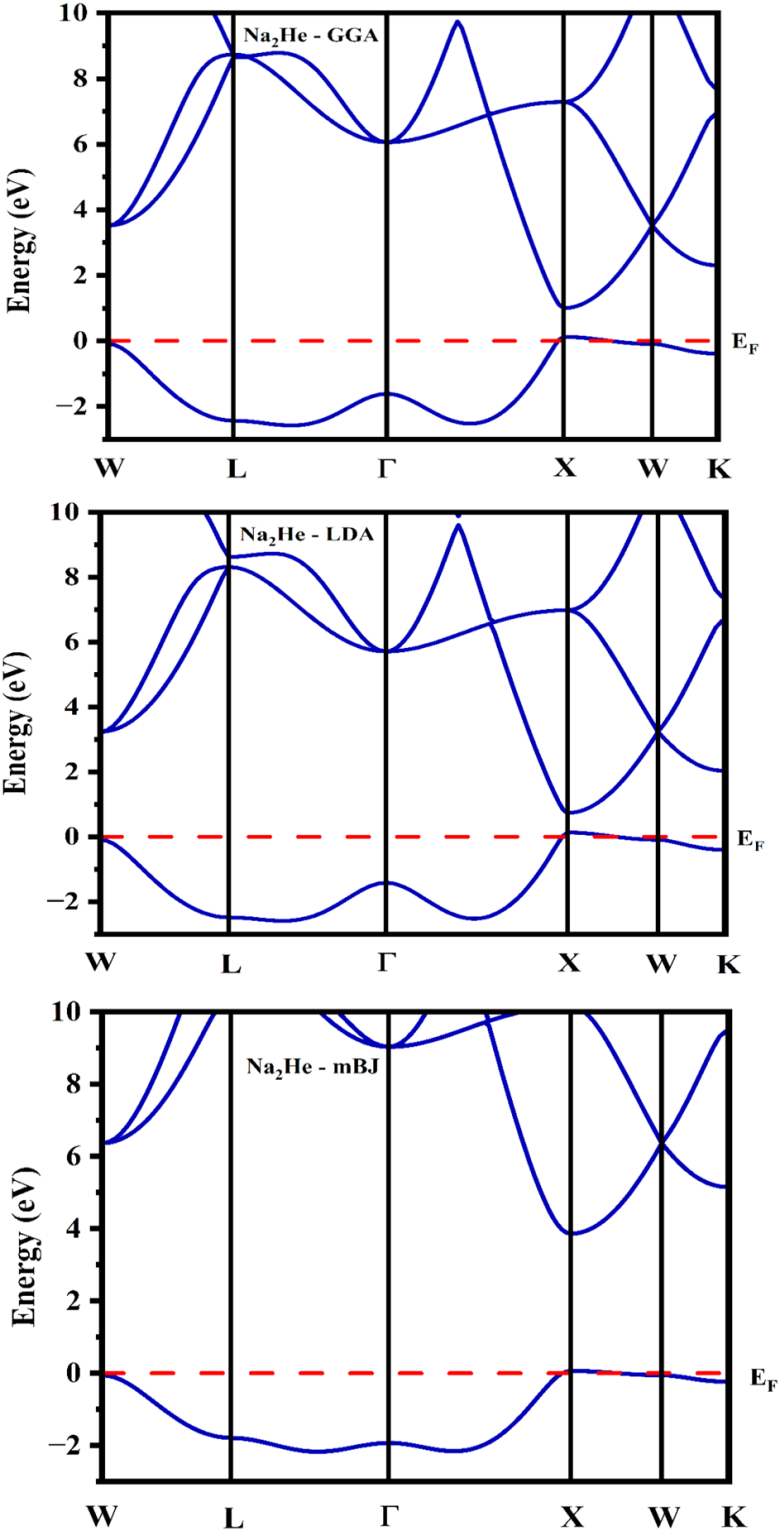
Band structure of Na_2_He.

**Figure 3:**
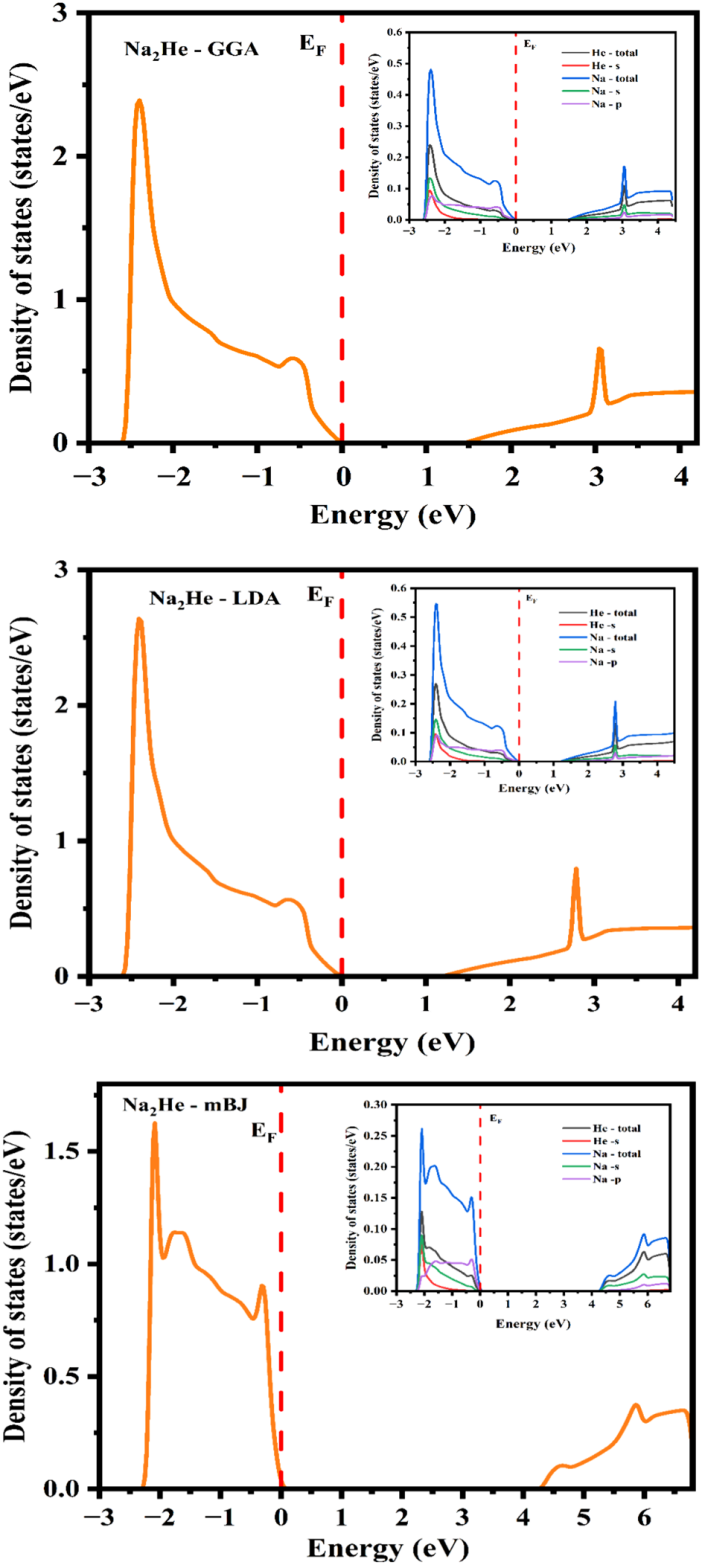
Total density of states for Na_2_He (inset: partial density of states).

Electronic band structure and DOS of disodium helide have been computed and are shown in Figs. 2 and 3. Nevertheless, no experimental study with respect to electronic properties for comparison. For the calculation of electronic properties such as band structure and DOS exchange correlation of GGA, LDA and mBJ were used and compared. The k path for band structure along high symmetry points of (W–L–Γ–X–W–K). In that, the Valence band maximum (VBM) and conduction band minimum (CBM) situates at X of k path. Since, the CBM and VBM lies at same k point, implying that the results of GGA and LDA exchange potentials reveals that Na_2_He is a direct bandgap semiconductor while the mBJ predicts direct bandgap insulator as shown in Fig. 2. DOS plot for GGA, LDA and mBJ were shown in Fig. 3. Based on the energy range of CBM and VBM, the band gap was calculated. The calculated bandgap values of disodium helide from the band structure and DOS is given in Table 1. Optical bandgap values were also listed in Table 1. which is same as electronic band gap, since the compound possess direct bandgap. It can be seen Partial DOS (inset) of GGA as shown in Fig. 3. that the highest occupied valence band arising from the Na *s*, *p* and He *s* states. The upper of conduction band is occupied by 2*s* and 2*p* states of Na. Similar Partial DOS profile has been observed for LDA and mBJ exchange correlations.

**Table 1:** Calculated bandgap and total energy of Na_2_He under various potentials.

Material	Exchange correlations	Bandgap E_g_ (eV)	Optical bandgap E_g_ (eV)	Total energy E_tot_ (Ry)
Na_2_He	GGA	1.455	1.455	− 654.26225
	LDA	1.185	1.185	− 651.39338
	mBJ	4.31	4.31	− 650.96687

### Transport properties

Based on the electronic property calculations, our projected compound Na_2_He is a semiconductor. As a result, I propose to execute excitonic effects. The effective mass is important in calculating excitonic effects. The effective mass is determined using the parabolic band approximation at the valence band maximum (VBM) and conduction band minimum (CBM). Table 2 shows the computed effective masses of electrons and holes. The effective mass can be calculated using the following relation,

**Table. 2:** The elucidated effective mass, exciton binding energy and exciton Bohr radius.

Compound	Electron	Hole	Exciton Binding energy E_b_ (meV)	Exciton Bohr radius ${{\varvec{a}}}^{\boldsymbol{*}}$ (Å)
Na_2_He	0.1512 ${m}_{e}$	0.3025 ${m}_{e}$	26.2	37.96

$$\frac{1}{{\mathrm{m}}_{\mathrm{eff}}}=\frac{1}{{\mathrm{\hbar }}^{2}}\frac{{\partial }^{2}\mathrm{E}}{\partial {\mathrm{k}}^{2}}$$

In the Na_2_He combination, the effective mass of holes is predicted to be larger than the electrons. Calculating exciton binding energy and exciton Bohr radius requires the combination of effective mass and the real portion of the dielectric function (static dielectric constant). The exciton binding energy and Bohr radius may be calculated using the following calculation^23–25^,

$${E}_{b} = 13.6\frac{{m}_{\mu }}{{m}_{0}}\frac{1}{{\varepsilon }_{1}{\left(0\right)}^{2}}$$

$${a}^{*} = {\varepsilon }_{1}(0)\frac{{m}_{0}}{{m}_{\mu }}{a}_{0}$$

The static dielectric constant is determined to be 7.233. Table 2 shows the predicted exciton binding energy and exciton Bohr radius. The predicted exciton Bohr radius is bigger than the optimized lattice parameters. As a result, our compound Na_2_He is classified as a Mott-Wannier type exciton.

### Optical properties

**Figure 4:**
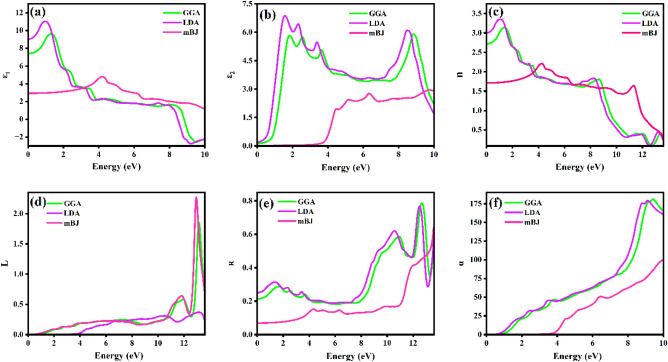
Calculated optical properties for 3D-Na_2_He.

On determining and studying the optical properties of a material, it aids to unveil the analogy of the material when it gets subjected or exposed to high energy photons. The optical properties suchlike refractive index n($\omega$), reflectivity R($\omega$), absorption coefficient $\mathrm{\alpha }\left(\omega \right)$, energy loss function L($\omega$) and complex dielectric function $\varepsilon (\omega )$ for the compound Na_2_He has been computed using PBE-GGA, LDA and mBJ exchange correlations via WIEN2k. The dielectric function is a property to study the interaction of the electron and photons dependent on the photon energy. The optical response for a compound at various photon energies can be determined via the dielectric function. The Fig. 4a and b represents the real and the imaginary parts of the complex dielectric function. The polarizability and the energy dissipation are analyzed by the real part (related with the material’s ability to store the electric energy) and imaginary part (related with the damping of the waves) of the dielectric function respectively. The real static points for the exchange correlations PBE-GGA. LDA and mBJ are 7.39, 9.02 and 2.93 eV, respectively and is highest at the point 1.32, 0.99 and 4.20 eV. The imaginary part of the dielectric function aids in understanding the optical bandgap and absorption. The results from PBE-GGA, LDA and mBJ shows that there is a frequency response for a miniature amount of energy. The imaginary part of dielectric function is highest at the point 1.84, 1.56 and 9.75 eV for PBE-GGA, LDA and mBJ respectively. The peaks observed at 1.84 eV corresponds to the inter bands observed in the band structure at 1.84 eV for GGA. The refractive index of a material elucidates the absorption and desorption of the photons by a material. The Fig. 4c depicts the curve between refractive index and the energy of the photon for PBE-GGA, LDA and mBJ. If the medium is rarer the refractive index holds a less value and vice-versa. Values of refractive indices vary with respect to the energy of the photon. The static refractive index values for GGA, LDA and mBJ are 2.72, 3.00 and 1.71 respectively. The curves of GGA and LDA are akin with different static point values. The n($\omega )$ increases when the photon energy increases and reaches the maximum at 3.14, 1.07 and 4.23 eV for GGA, LDA and mBJ and gradually decreases. The Fig. 4d illustrates the energy loss function versus photon energy curve. The amount of energy that the compound losses with respect to the increase in the photon energy is specified by the energy loss function. In the band gap of the compound there is no loss in the energy. The utmost energy loss is acquired at 13.16, 12.94 and 13.12 eV for PBE-GGA, LDA and mBJ respectively. Those peak values in the energy loss function corresponds to the plasma resonance, defined to be plasma frequency^26^. Reflectivity by term means the ability of a material to reflect the incident light. The Fig. 4e illustrates the reflectivity curve of Na_2_He for PBE-GGA, LDA and mBJ exchange correlations. The material starts to reflect light at the points 0.21, 0.25 and 0.06 eV respectively. The GGA and LDA curves are similar as the former underestimates and the latter overestimates the values. The curves smoothly increase with the increase in the photon energy. To comprehend the amount of light that gets absorbed by a material in a unit length, the absorption coefficient should be studied. In Fig. 4f it can be observed that the absorption by the compound Na_2_He even for a miniature energy. The absorption starts from 1.45 eV and better absorption in 2 eV which indicates the absorption in the visible region and the peak increases to ultra-violet region. The absorption by the compound gradually accelerates and is maximum at 9.4 and 9.13 for PBE-GGA and LDA respectively, when the photon energy boosts.

### Na ***K*** edge in Na_2_He for XANES

**Figure 5:**
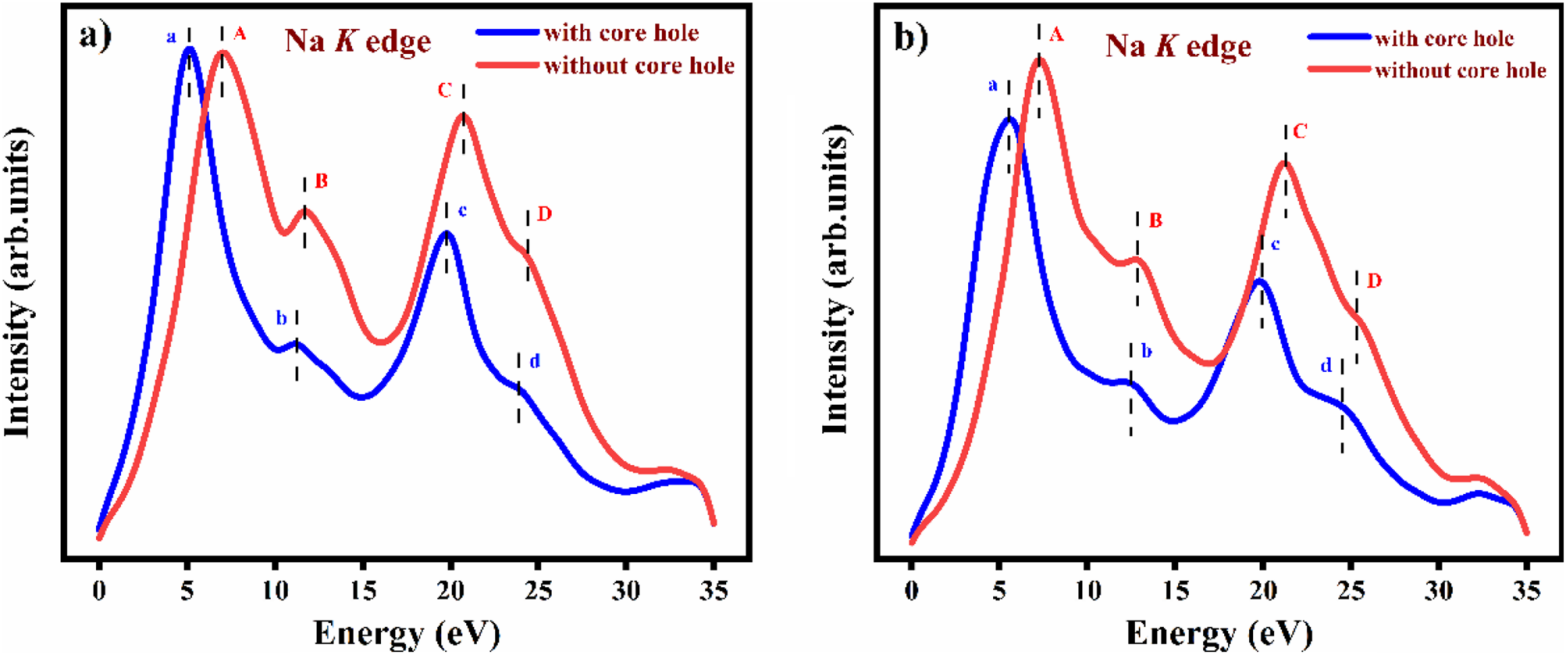
Theoretical calculation of sodium *K* edge XANES in Na_2_He with and without core hole, (**a**) For 2 × 2 × 2 supercell, and (**b**) For 3 × 3 × 3 supercell.

The theoretical calculations of the Na *K* edge absorption spectra are represented in Fig. 5. I have introduced, for the first time, the theoretical spectroscopic calculation for the Na_2_He compound. Unfortunately, there is no experimental evidence available for this compound. Na *K* edge x-ray absorption near edge structure (XANES) provides insights into electronic transitions and the local atomic environment of sodium atoms in Na_2_He. By measuring the X-ray absorption at various energies near the Na *K* edge, the technique can reveal details about the oxidation state, coordination number, and bonding characteristics of sodium in Na_2_He. However, it’s important to note that Na_2_He is not stable under ambient condition. Therefore, I focus our discussion solely on the absorption peaks, intensity and energy of the Na *K* edge spectra.

To perform the sodium (Na) *K* edge XANES calculations, I utilized 2 × 2 × 2 and 3 × 3 × 3 face centred cubic supercells based on the existing literature for various compounds^27–30^. In this discussion, our primary focus is on the behaviour of the Na 1*s* core electron *(*$s\to p$ electronic transitions) with and without considering the core hole electron in the Na_2_He compound.

I begin by examining the 2 × 2 × 2 supercell. From Fig. 5a, I observe four spectral features denoted as a, b, c, and d when considering the core hole effect, and A, B, C, and D when disregarding the core hole effect. These four spectral features, or absorption peaks, have corresponding values at around 7.20 eV, 11.98 eV, 21.10 eV, and 24.32 eV in the absence of the core hole effect. When the core hole is present, the absorption peaks exhibit a slight blue shift, as depicted in Fig. 5a.

Turning our attention to the 3 × 3 × 3 supercell, I notice that the absorption peaks at certain positions, such as b and d, are sharper when the core hole effect is present compared to the 2 × 2 × 2 supercell, as shown in Fig. 5b. However, in the absence of the core hole, the absorption peak at position B is slightly less sharp compared to the 2 × 2 × 2 supercell. This difference can be attributed to the increased accuracy of spectroscopic calculations as the supercell size grows.

Indeed, the choice of supercell size can have implications for the physical properties of Na_2_He. Larger supercells provide a more accurate description of its electronic structure, bonding, and spectroscopic calculations. This, in turn, can impact predictions related to its stability, phase transitions, and other properties. The 3 × 3 × 3 supercell contains more atoms and more effectively replicates the crystal lattice than the 2 × 2 × 2 supercell, a critical consideration when studying bulk properties of materials like Na_2_He, as it ensures a better representation of the true crystal structure and reduces finite-size effects.

Moreover, increasing the supercell size reduces the interaction between neighbouring core hole electrons. In the absence of the core hole, the energy values for the four spectral features are approximately 7.50 eV, 13.08 eV, 21.44 eV, and 25.76 eV in the 3 × 3 × 3 supercell, all of which are higher compared to the 2 × 2 × 2 supercell. When considering the core hole effect, I notice that the intensity is lower and suppressed in the absorption peak A in the 3 × 3 × 3 supercell compared to its absence. Additionally, in the absence of the core hole, the energy value in absorption peak C is slightly blue-shifted towards lower photon energy compared to when the core hole effect is present in the 3 × 3 × 3 supercell.

### 2D-Na_2_He dynamic stability

**Figure 6:**
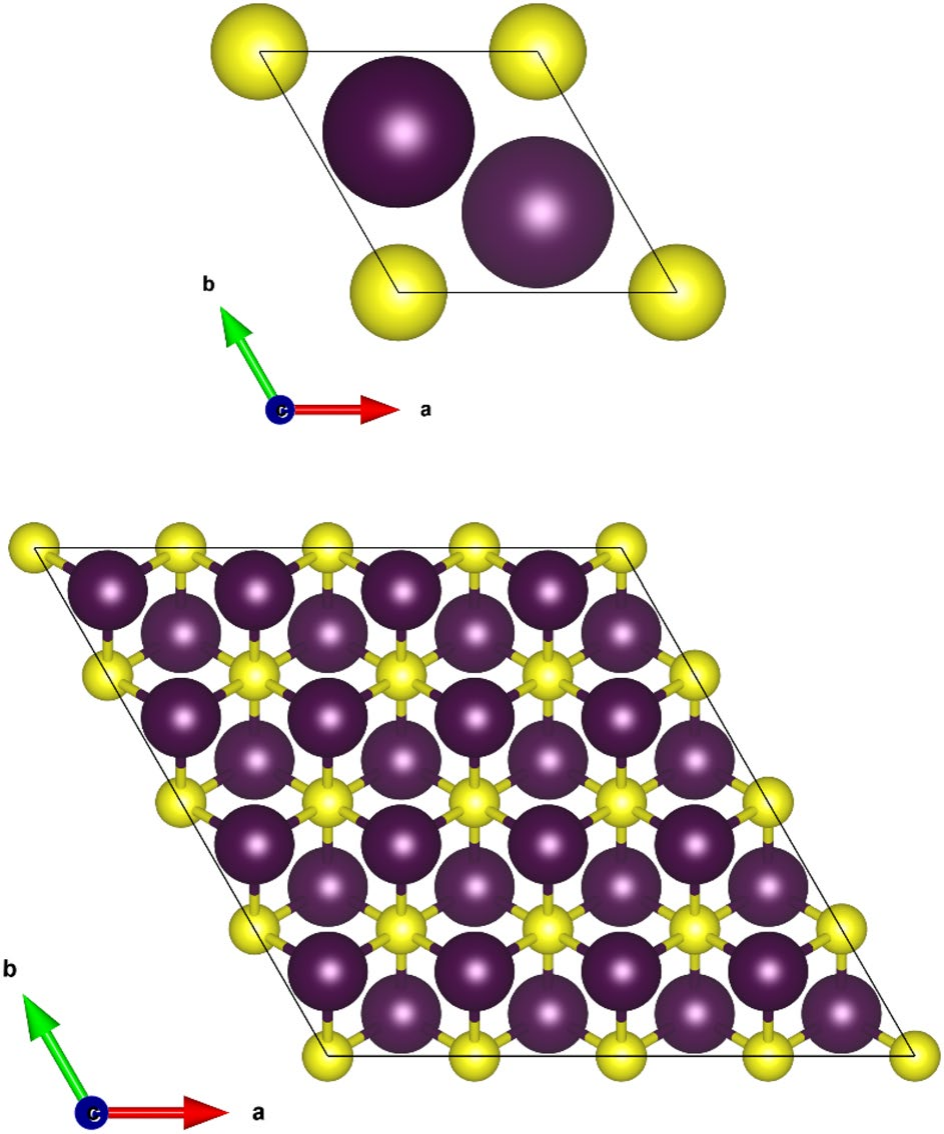
Unit and supercell structure for 2D-Na_2_He.

**Figure 7:**
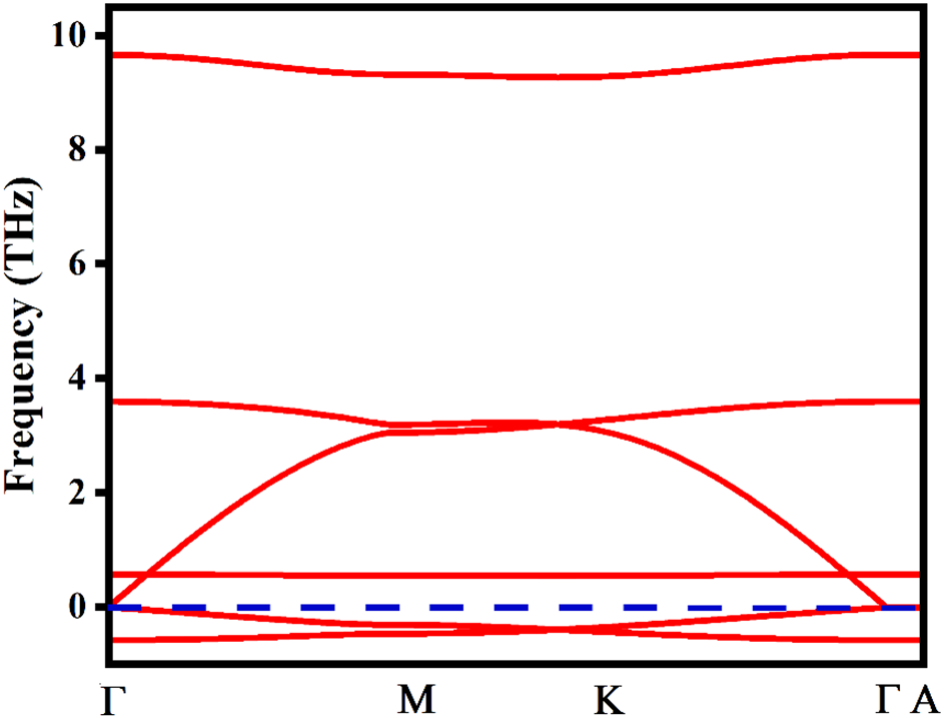
Phonon band structure for 2D-Na_2_He.

The two-dimensional electronic structure of Na_2_He formed from a chemically inert element and an alkali metal possesses a trigonal structure in which a Na atom occupies the center of the trigonal face surrounded by Na and He atoms. The compound crystallizes in the space group P3m1-164. The 3D structure of Na_2_He is reported to be stable at high pressures^31,32^. The 3D-Na_2_He compound is unstable (without pressure) which emanated us to study the compound’s properties in two-dimension via its monolayers. The Fig. 6a portrays the 1 × 1 × 1 unit cell of 2D-Na_2_He and the Fig. 6b depicts the 4 × 4 × 1 super cell of the monolayer where the structure is alike the 1T-Na_2_S^33^. To study the 2D structure, its existence and the so far stability of the compound 2D-Na_2_He the phonon calculations were performed and as shown in Fig. 7. The phonon calculations via the Phonopy interface with WIEN2k resulted that the free-standing Na_2_He monolayer is unstable with negative phonon modes. The monolayer is dynamically unstable and is not quenchable to ambient conditions^34^.

## Conclusion

The electronic structure and ground-state properties of Na_2_He are calculated in the present work using the first principles method. The total energy calculations and electronic properties were studied using different exchange potentials. From the band structure, GGA and LDA predicted bandgap of Na_2_He is about 1.455 and 1.185 eV, while from mBJ it is about 4.31 eV. But the total energy calculated from self-consistent field indicates that GGA provides lower ground state energy compared to LDA and mBJ. Hence, GGA provided results is promising. Thus, disodium halide is a direct bandgap semiconductor with a gap of 1.455 eV.

The optical properties for the cubic Na_2_He were studied. Effective mass, and excitonic properties were studied and the results implies that the compound possess Mott-wannier type exciton. Based on absorption in the UV–visible region and refractive index implies the material can be utilized in optoelectronic devices. Na K edge XANES for 2 × 2 × 2 and 3 × 3 × 3 supercells were computed and clearly indicates that the 3 × 3 × 3 supercell is more accurate than 2 × 2 × 2 supercell due to increasing number of atoms. In order to examine the possibility of the material to form as a monolayer, its dynamical stability of 2-D structure of Na_2_He compound were analyzed and the results provide that the compound is dynamically unstable which may be due to the calculations were done under ambient conditions.

## Data Availability

The datasets used and/or analysed during the current study available from the corresponding author on reasonable request.
